# Factors associated with the use of UK emergency services in children and young people with intellectual developmental disabilities

**DOI:** 10.1186/s12916-026-05277-x

**Published:** 2026-10-28

**Authors:** Irene O. Lee, Jeanne Wolstencroft, Harriet Housby, Marianne B. M. van den Bree, Samuel J. R. A. Chawner, Jeremy Hall, Michael J. Owen, IMAGINE ID Consortium, David H. Skuse, Jeanne Wolstencroft, Irene O. Lee, Samuel Chawner, Jeremy Hall, Josh Hope-Bell, Michael J. Owen, Marianne van den Bree, F. Lucy Raymond, Ramya Srinivasan, David H. Skuse

**Affiliations:** 1 Paediatric Mental Health Sciences Centre, Great Ormond Street Institute of Child Health, University College London, London, UK; 2 Department of Psychology, College of Health Medicine and Life Sciences, Brunel University of London, London, United Kingdom; 3 The Division of Psychology and Language Sciences, Faculty of Brain Sciences, University College London, London, UK; 4 Centre for Neuropsychiatric Genetics and Genomics, Division of Psychological Medicine and Clinical Neurosciences, Cardiff University, Cardiff, UK; 5 Neuroscience and Mental Health Innovation Institute, Division of Psychological Medicine and Clinical Neurosciences, Cardiff University, Cardiff, UK; 6 IMAGINE ID Consortium, London, UK

**Keywords:** Intellectual developmental disabilities, Emergency service, Multimorbidity, Inequality, Emotional and behavioural adjustment.

## Abstract

**Background:**

Children and young people (CYP) with intellectual developmental disabilities (IDD) experience substantial health needs and higher healthcare utilisation compared with the general paediatric population. However, factors affecting the emergency healthcare use among children with IDD of genetic aetiology remain poorly understood. This study aimed to examine demographic, socioeconomic, behavioural, genetic, and clinical factors of emergency service use among CYP with IDD in England.

**Methods:**

This retrospective cohort study used linked administrative healthcare data from Hospital Episode Statistics (HES) emergency service records obtained from National Health Service (NHS) England. Participants were CYP aged between 4 and 15.5 years with confirmed pathogenic genetic variants associated with IDD identified via NHS medical genetic clinics. The outcome was the yearly emergency service use rate, calculated as the total number of lifetime emergency visits divided by age (years). Independent variables included demographic and socioeconomic factors, genetic characteristics, region of residence, adaptive functioning, behavioural and emotional adjustment, prosocial behaviour, general health status, and system-specific medical conditions. Primary caregivers completed questionnaires and provided information regarding the child’s medical history and demographic information.

**Results:**

The average emergency attendance rate in this cohort was 0.73 visits per annum, which was much higher than that among children in the same age range from the general population. Emergency services were used more by younger IDD children with poorer adaptive functioning, more externalising and less prosocial behaviour, as well as those with complex multisystem conditions. Frequent attendance was associated with high rates of medical comorbidities especially seizures and respiratory problems. Regional differences were found with relatively higher usage in London and the North East of England. Attendance rates were not influenced by sex, ethnicity, socioeconomic deprivation, genetic variant type or inheritance status.

**Conclusions:**

This novel study demonstrated emergency healthcare utilisation among CYP with IDD of known genetic aetiology in England was primarily associated with clinical complexity, functional impairment, and younger age rather than socioeconomic or genetic factors. Their vulnerability to multisystem morbidity as well as regional disparities highlight the need for improved coordinated care and targeted service planning to reduce emergency healthcare dependence among children with disabilities.

**Supplementary Information:**

The online version contains supplementary material available at https://doi.org/10.1186/s12916-026-05277-x.

## Background

Intellectual developmental disability (IDD) is characterised by significantly impaired intellectual functioning and deficits in adaptive behaviours [1–3]. IDD affects 2–3% of population worldwide [4, 5] and is the most common developmental disorder [1]. In the UK, children and young people (CYP) with IDD have higher rates of multimorbidity, unmet healthcare needs, and preventable hospital utilisation than typically developed children [6, 7]. As a group, they are affected by relatively high levels of physical, neurological, and behavioural disorders, compared to the general paediatric population, which can complicate healthcare management across the life course [8].

Emergency service use is widely regarded as a sensitive indicator of unmet need, fragmented care, or insufficient access to timely community-based services. Evidence from large North American administrative datasets indicates that children with IDD visit emergency departments at approximately 1.8 times the rate of those without developmental disabilities, leading to higher service frequency, increased costs, and greater overall demand on emergency resources [9]. In England, children with learning disabilities also experience relatively higher rates of emergency department attendance and unplanned hospital admission than peers without disabilities [10, 11]. Data derived from National Health Service (NHS) England administrative sources indicate potentially preventable emergency care utilisation is disproportionately high among individuals with complex neurodevelopmental conditions [10].

Challenging behaviours and mental health crises are common reasons for emergency service visits among CYP with developmental disorders and often reflect unmet needs in community and outpatient care. Difficulties with communication add to the clinical burden that is placed upon acute care services [8, 12]. Parents and caregivers of affected children frequently report that the environment at emergency services is poorly adapted to their child’s communication and sensory needs. This can impair decision-making and foster patterns of repeat emergency utilisation [8]. Within the IDD population, poor general health, seriously impaired adaptive functioning, behavioural difficulties and psychosocial stress are associated with greater emergency care utilisation [13, 14]. Variance in rates of emergency service attendance have been linked to differences in local healthcare accessibility, resource availability and social determinants of health, underscoring the importance of the environmental context when comparing service use patterns across the UK [15].

This novel longitudinal study focused on children and young people with genetically confirmed causes of IDD. Our cohort was collected nationally in the UK, recruited via the National Health Service (NHS) Regional Genetic Services. They were studied over a period of 10 years, from 2015 to 2025 [16]. Participants comprised a heterogeneous group with a diverse range of genetic conditions, including single gene disorders and copy number variants. In some cases, the genetic abnormality was inherited. We investigated the possibility that carriers could have limited ability to utilise healthcare services for their child. To date, evidence on that subject remains limited, but genome sequencing technology is becoming more widely available for children with complex developmental presentation and their family members. We aimed to discover whether early genetic diagnosis might guide interventions that would limit the need for families to attend emergency services.

Family factors, such as household income, general health status, and family support networks have also been associated with the likelihood of emergency care utilisation in children with disabilities, indicating that social as well as clinical factors contribute to service use [2]. Area-level deprivation, commonly measured using the Index of Multiple Deprivation (IMD), is strongly associated with higher emergency healthcare utilisation across paediatric populations in England [17]. Despite this recognised vulnerability, evidence specifically examining the interaction between socioeconomic deprivation and emergency healthcare utilisation in CYP with IDD remains limited. Regional variation in emergency service use has also been documented, with London and parts of Northern England demonstrating relatively elevated attendance rates compared with the general population [18].

With legal guardians’ permission, we linked data collected on our cohort’s medical, social and mental health histories to administrative hospital data from NHS England Hospital Episode Statistics (HES), including Accident & Emergency (A&E) and Emergency Care Data Set (ECDS) components. We investigated potential influences on emergency service usage, including age, general health status, adaptive functioning, behavioural difficulties, socioeconomic status (including ethnicity, sex, area-level deprivation) and region of residence. We selected these factors because they have been linked to increased usage of such services in other populations and we hypothesised that all these covariates could influence the outcome of the emergency service utilisation.

## Methods

### Study participants

Our longitudinal study was entitled “Intellectual Disability and Mental Health: Assessing the Genomic Impact on Neurodevelopment (IMAGINE-ID)”. We recruited 3407 UK participants [16], largely from 23 UK NHS Regional Genetics Centres, between 2015 and 2020. All were over 4 years of age at enrolment and had received standardised molecular genetic testing [19]. Pathogenic variants were classified according to the American College of Medical Genetics and Genomics guidelines [20]. Participants were followed up in a further study between 2020 and 2025 [21, 22].

The study was approved by the London Square Research Ethics Committee in the UK. The legal guardians of the participants provided consent to link the study data with NHS clinical records. All methods were carried out in accordance with relevant guidelines and regulations.

### Data source

We linked study participation records to administrative healthcare data from NHS Hospital Episode Statistics (HES) Accident and Emergency (A&E) and Emergency Care Data Set (ECDC) records. These were available for the period from January 2007 to April 2022. HES captures all NHS-funded hospital activity in England. This period included lifetime records of emergency service visits for our participants from birth up to 15.5 years of age. A&E and ECDC datasets were combined according to the individual identifier and the variable “Arrival date” of both datasets; duplicates were removed. We calculated an emergency service use rate (dependent variable) by taking the total lifetime emergency visits of each individual and dividing by their age in years. This gave us a mean attendance rate per annum, standardised for age.

Potential influences on emergency attendance rate that we measured included:

1. Genetic factors
  1.1 Variant type [Single Nucleotide Variant (SNV) or Copy Number Variant (CNV)],
  1.2 Genetic inheritance (familial as inherited variant or *de novo*).

Genetic information from the genetic centres indicated participants had either a putatively pathogenic copy number variant (CNV) or single nucleotide variant (SNV). Overall, 629 participants (78.6%, see Table 1) had a CNV and 171 (21.4%) had a SNV. We obtained information about the potential parental origin of the abnormality from 530 participants (66.3%). Inherited, familial status, was assigned when a variant was inherited from the mother or father. *De novo* status was assigned when parental DNA testing showed the variant was not carried by either parent. 327 (61.7%) CYP participants with information on inheritance had *de novo* variant and 203 (38.3%) had a familial variant.

2. Neurodevelopmental and behavioural measures
  2.1 Adaptive Behaviour Assessment System 3 (ABAS-3): General Adaptive Composite (GAC) standard score,
  2.2 Strength and Difficulties Questionnaire (SDQ): total and subscale scores.

The Adaptive Behaviour Assessment System 3 (ABAS-3) was used to evaluate daily living skills [23]. Caregivers of 451 participants (56.4%) completed the ABAS-3 questionnaires online or with an interviewer on the phone. General Adaptive Composite (GAC) standard scores from ABAS-3 were calculated. According to the ABAS-3 scoring rules, GAC standard scores lower than 70 are regarded as ‘Extremely Low’, between 71 and 80 as ‘Low’, 81–90 as ‘Below Average’, 91–110 is the ‘Average’ range, 111–120 as ‘Above Average’ and higher than 120 is ‘High’ [23].

Caregivers of 738 participants (92.3%) completed the Strengths and Difficulties Questionnaire. The SDQ is widely used to screen for emotional and behavioural adjustment in population surveys [24], and is suitable for evaluating emotional and behavioural difficulties in CYP with IDD [25]. It comprises four problem domain sub-scales: emotional symptoms; conduct problems; hyperactivity, impulsivity and inattention difficulties; and peer relationship problems. There are also scales for prosocial behaviour. The four problem domain scales are combined to yield a total difficulties score [24, 26]. SDQ total difficulties scores have been categorised into four bands based on general population survey data [see Additional File 1: Note S1 [24, 26–29] ]: 80% of UK children score in a ‘close to average’ range, regarded as ‘normal’; 10% score in a ‘slightly raised’ range of problems, regarded as ‘borderline’; and 10% score in the ‘high’ or ‘very high’ range, indicative of problems of potential clinical significance [29]. We had permission to use the ABAS-3 and SDQ questionnaires for this study.

3. Health status
  3.1 General health condition,
  3.2 Presence of system-specific disorders (14 categories).

Caregivers completed a bespoke medical history questionnaire, which captured information about the child’s general health, developmental attainments and medical conditions. They also rated their opinion about their child’s general health on a 5-point Likert scale from “Very Bad” to “Very Good”. The medical history questions for 14 system-specific health problems are listed in Additional File 1: Note S2.

4. Demographic and socioeconomic factors
  4.1 Age (years),
  4.2 Sex,
  4.3 Ethnicity,
  4.4 Index of multiple deprivation (IMD) decile,
  4.5 Region of residence (England regions).

Caregivers provided demographic and socioeconomic information. Individual postcodes were used to ascribe family homes to an index of multiple deprivation (IMD) decile (1 = most deprived, 10 = least deprived) according to the UK Office for National Statistics (ONS) [30]. Home addresses were categorised into the nine regions of England specified by the UK ONS [31].

### Statistical analysis

Descriptive statistics were computed for all variables. Due to skewness, the dependent variable (emergency attendance rate) was transformed to approximate a normal distribution. Correlation analyses were employed to examine the associations between continuous independent variables (age, GAC, SDQ scores) and dependent variable. We used parametric statistics (*t*-test and ANOVA analyses) to identify the impact of categorical independent variables including demographic, socioeconomic, genetic, region of domicile and health-related variables on the emergency use rate. To ensure robustness against false positives across multiple comparisons, the Benjamini-Hochberg procedure was applied, providing a more powerful alternative to Bonferroni correction while controlling the false discovery rate. A series of linear regression models were conducted to examine the associations between these variables and the emergency utilisation rate. Statistical significance was set at *p* < 0.05. Data analyses were performed in SPSS version 28 on the University College London Data Safe Haven platform.

## Results

We analysed lifetime emergency department data for the years 2007 to 2022, provided by NHS England for 800 participants from our cohort. This subset of participants was aged between 4 and 15.5 years at the time of recruitment into IMAGINE-ID project (mean age ± SD = 11.4 ± 2.5 years; 59% male; see Table 1).

**Table 1:** Participant demographic information

Demographic category	Frequency, *N* (% of total count)
**Sex**	
Male	468 (58.5%)
Female	332 (41.5%)
Total count	800
**Age at recruitment** (years)	
Mean ± SD [95% CI]	11.4 ± 2.5 [11.2–11.6]
Median	11.5
Range	4.0-15.49
**Ethnicity**	
White	700 (87.5%)
Asians	29 (3.6%)
Black	< 10
Mixed	38 (4.8%)
Unknown	< 10
**Variant Type**	
SNV	171 (21.4%)
CNV	629 (78.6%)
**Genetic Inheritance**	
* De novo*	327 (40.9%)
Familial	203 (25.4%)
Unknown	270 (33.8%)
**Index of Multiple Deprivation**	
1 (most deprived)	75 (9.4%)
2	93 (11.6%)
3	72 (9.0%)
4	56 (7.0%)
5	67 (8.4%)
6	99 (12.4%)
7	76 (9.5%)
8	78 (9.8%)
9	87 (10.9%)
10 (least deprived)	93 (11.6%)
Missing data	4 (0.5%)
**Region of England**	
North East	29 (3.7%)
North West	69 (8.7%)
Yorkshire and the Humber	110 (13.9%)
East Midlands	60 (7.5%)
West Midlands	113 (14.3%)
East England	115 (14.6%)
London	77 (9.7%)
South East	147 (18.6%)
South West	70 (8.9%)
Missing data	10 (1.3%)

Note: *N*=number of cases; *CI*=Confidence Interval; *SD*=standard deviation; *SNV*=Single Nucleotide Variant: *CNV*=Copy Number Variant; *De novo*=non-inherited variant; *Familial*=inherited variant

The average emergency visit rate in our cohort was 0.73 visits per annum (SD = 0.75, 95% CI = 0.68–0.78). For comparison, a Nuffield Trust report of emergency A&E attendances for UK children under 15 years old found a rate of 0.475 visits per annum [32]. In neither case is the attendance rate corrected for repeat attendees.

### 1. Influences on emergency service use 

#### *Age*

**Fig. 1:**
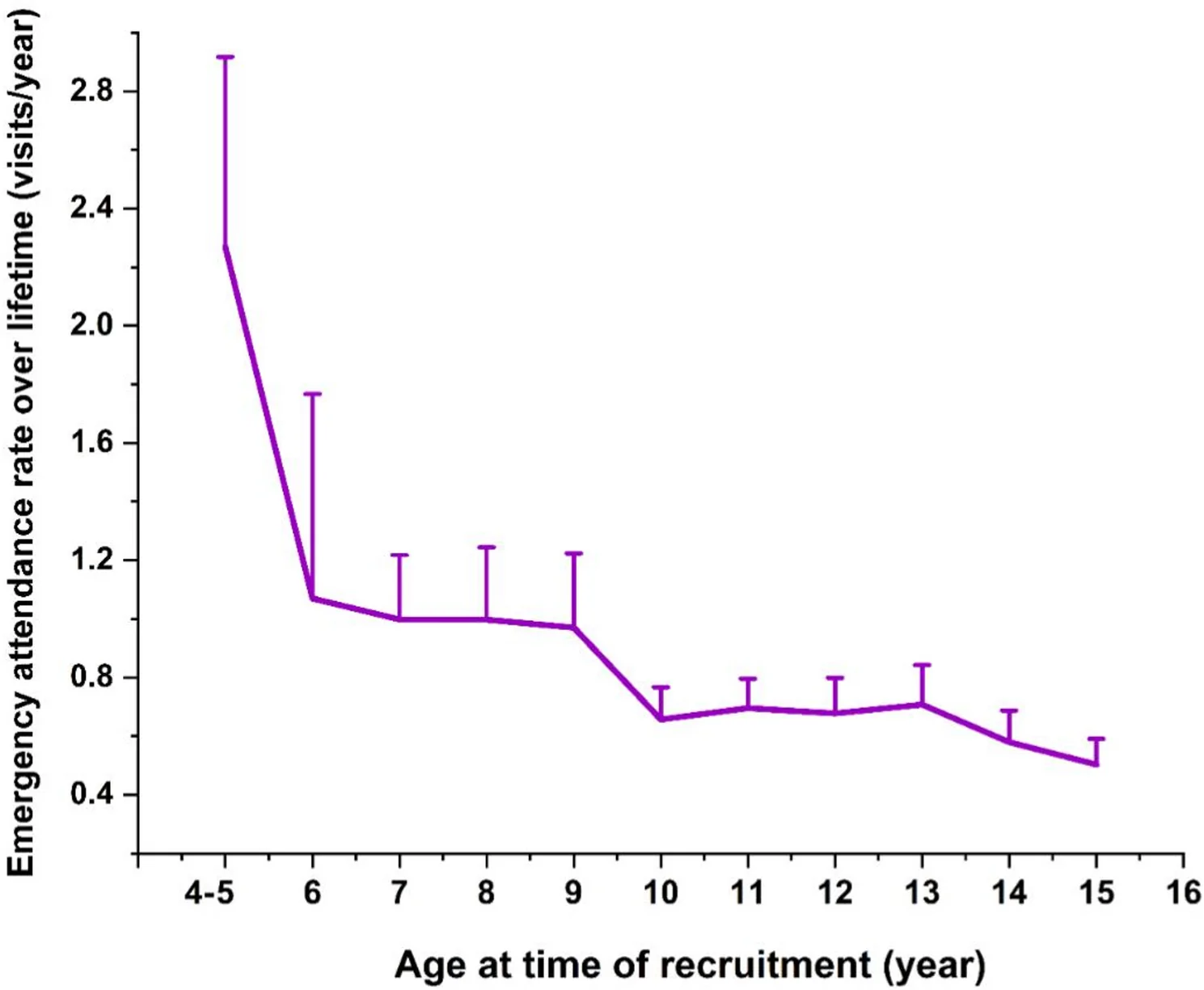
The impact of age on emergency attendance rate in children and young people with intellectual developmental disabilities (with 95% confidence interval error bars)

In the IMAGINE-ID cohort, age was significantly negatively correlated with emergency use rate (*r*=-0.237, *p* < 0.001, *N* = 800, see Table 2). Younger children had higher annualised emergency attendance rates compared with older children (*F* = 5.958, *η*^2^ = 0.070, *p* < 0.001, see Fig. 1). Emergency department visits among our participants aged under 5 years averaged 2.27 visits per annum (see Fig. 1). For comparison, emergency service visits per annum for children aged 0 to 4 years in the general population are in the range 0.60 to 0.80 visits per annum [see Additional File 1: Table S1 [33, 34] ]. IMAGINE-ID participants aged 5 to 15 years made between 0.50 and 1.07 visits per annum compared with the general population figure of 0.18 to 0.25 visits per annum for those aged 5 to 14 years.

**Table 2:** Correlations between the emergency use rate and independent variables

Variable		Emergency service use rate (visit/yr)	
		Without control variable ^a^	Control for age/gender ^b^
**Age**	Correlation	-0.224	---
	*p*-value	*< 0.001*	*---*
	df	736	---
**GAC standard score**	Correlation	-0.121	-0.122
	*p*-value	*0.010*	*0.010*
	df	449	447
**Total difficulties score**	Correlation	0.094	0.103
	*p*-value	*0.010*	*0.005*
	df	736	734
**Emotional symptoms score**	Correlation	0.030	0.067
	*p*-value	0.408	0.071
	df	736	734
**Conduct problems score**	Correlation	0.091	0.065
	*p*-value	*0.013*	0.079
	df	736	734
**Hyperactivity score**	Correlation	0.079	0.089
	*p*-value	*0.033*	*0.015*
	df	736	734
**Peer problems score**	Correlation	0.073	0.072
	*p*-value	0.050	0.050
	df	736	734
**Prosocial score**	Correlation	-0.136	-0.098
	*p*-value	*< 0.001*	*0.008*
	df	736	734

Df=degree of freedom; ^**a**^=zero-order (Pearson) correlations; ^**b**^=Correlations controlling for age and gender

#### *Adaptive functioning*

The average General Adaptive Composite (GAC) standard scores of this cohort were 63.5 ± 14.3 (95% CI = 62.2–64.8). Scoring rules from the ABAS-3 place this in the “Extremely Low” range of daily adaptive ability. Lower GAC scores, which represented poorer adaptive ability, were significantly associated with more frequent emergency service use (*r*=-0.121, *p* = 0.010, Table 2) and the association still stands controlling for their age and gender (*r*=-0.122, *p* = 0.010).

#### *Behavioural characteristics*

The mean of the total difficulties score from the SDQ was 20.3 ± 6.8 (95% CI = 19.8–20.8, see Additional File 1: Table S2) which is classified in the “Very High” band; whereas that of conduct problems and hyperactivity scores were 3.5 ± 2.4 (95% CI = 3.3–3.7) and 7.9 ± 2.1 (95% CI = 7.7–8.1) respectively, which are both categorised in the “Slightly Raised” bands. These total and subscale scores were positively correlated with emergency attendance rate (*r* between 0.079 and 0.094, *p*-values < 0.033). Higher levels of total difficulties and hyperactivity were associated with higher emergency attendance rates independent of the participant’s age and gender (*r* = 0.103 and 0.089 respectively, both *p*-values < 0.015).

Prosocial behaviour scores averaged 5.2 ± 2.9 (95% CI = 5.0-5.4). This places them in the “Very Low” band and were negatively correlated with emergency use rate (*r*=-0.136, *p* < 0.001 and *r*=-0.098, *p* < 0.008 controlling for age and gender). Lower prosocial functioning was associated with higher emergency attendance rates. Other two SDQ subscale scores were not independently significant (*r* between 0.030 and 0.073, *p*-values > 0.05). The average scores and band classifications of the other subscales are listed in the Additional File 1: Table S2.

#### 2. General health

**Fig. 2:**
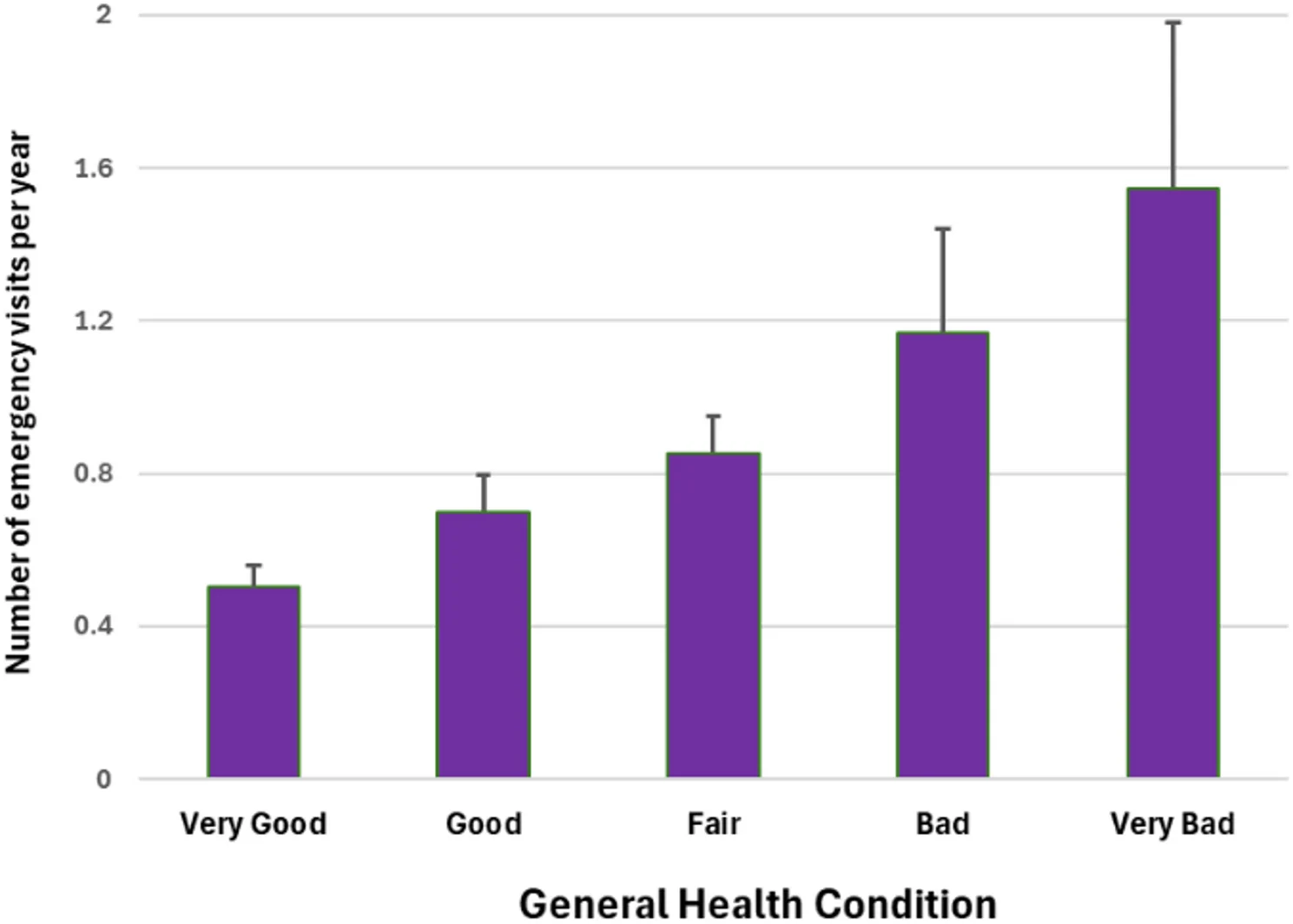
Emergency utilisation rates among cohort participants across varying degrees of general health. Error bars represent 95% confidence intervals

Children with more severe general health problems made relatively greater use of emergency services (df = 716, *F* = 17.022, η^2^ = 0.087, *p <* 0.001, see Fig. 2). Across the full age-range, those with “Very Bad” general health made 1.6 (95% CI = 0.4-2.0) visits per annum, over three times as frequently as those with “Very Good” health [0.5 (95% CI = 0.06–0.6) visits per annum].

#### 3. System-specific health problems

**Fig. 3:**
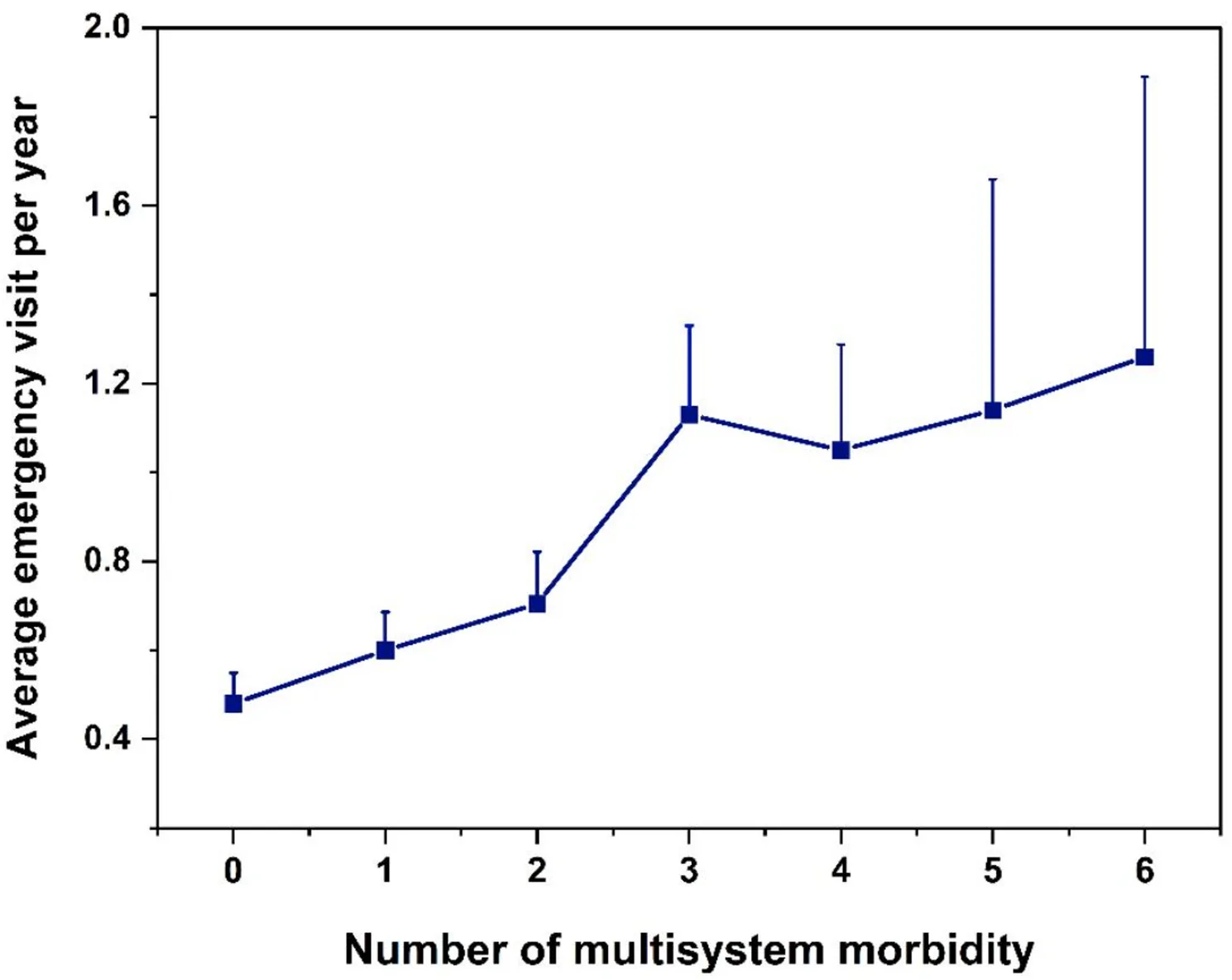
Emergency visit rates for children with multisystem morbidities (with 95% confidence interval error bars)

Considering information about health problems associated with specific body systems, there was a higher usage of emergency healthcare service in those with seizures (*t*=-5.529, *d* = 0.586, *p* < 0.001, see Table 3), chest or respiratory problems (*t=*-5.960, *d* = 0.561, *p* < 0.001), cardiac conditions (*t*=-4.472, *d* = 0.490, *p* < 0.001), gastrointestinal problems (*t=*-3.215, *d* = 0.307, *p* = 0.001), endocrine or hormonal disorders (*t*=-2.471, *d* = 0.293, *p* = 0.014) and musculoskeletal problems (*t=-2.541*, *d* = 0.323, *p* = 0.011). Many participants had multisystem morbidities, and those were associated with greater emergency healthcare utilisation (*F* = 12.490, *η*^2^= 0.138, *p* < 0.001, see Fig. 3). Specific information about the relationship between morbidity types and emergency healthcare usage is provided in Additional File 1: Table S3.

**Table 3:** Emergency visits per annum among the cohort participants with specified health-related conditions

Health related disorder*	Proportion affected	Emergency visits per annum				
		Without disorder^a^	With disorder^b^	*t*	*d*	*p*-value
Seizures	124 (28.2%)	0.61	0.98	-5.529	0.586	*< 0.001*
Respiratory	189 (40.4%)	0.59	0.92	-5.960	0.561	*< 0.001*
Cardiac	109 (23.6%)	0.65	0.96	-4.472	0.490	*< 0.001*
Gastrointestinal	182 (39.9%)	0.66	0.84	-3.215	0.307	*0.001*
Growth/Hormone	89 (20%)	0.68	0.87	-2.471	0.293	*0.014*
Bone/Skeletal	74 (16.3%)	0.68	0.91	-2.541	0.323	*0.011*
Genital	70 (15%)	0.70	0.84	-2.091	0.271	0.054
Cranial	12 (2.6%)	0.62	1.05	-1.866	0.546	0.063
Sleep	318 (67.7%)	0.66	0.76	-1.851	0.182	0.065
Muscle/Movement	293 (64.3%)	0.65	0.76	-1.661	0.162	0.097
Auditory	168 (36.6%)	0.70	0.78	-1.507	0.146	0.132
Cleft palate	23 (4.9%)	0.72	0.85	-1.121	0.240	0.263
Vision	268 (57.4%)	0.72	0.73	-0.962	0.090	0.336
Renal	33 (7.1%)	0.72	0.73	-0.309	0.056	0.758

Note: * The medical history questionnaire is described in Additional File 1: Note S2. Caregivers answered “yes” or “no” to a series of questions about system-specific health related disorders. Visits to emergency services were not necessarily related to that disorder on every occasion. Without disorder^a^ =the group without specific health-related problem; With disorder^b^=the group with specific health-related problem. *d*=Cohen’s *d*

#### 4. Geographical regional variation

**Fig. 4:**
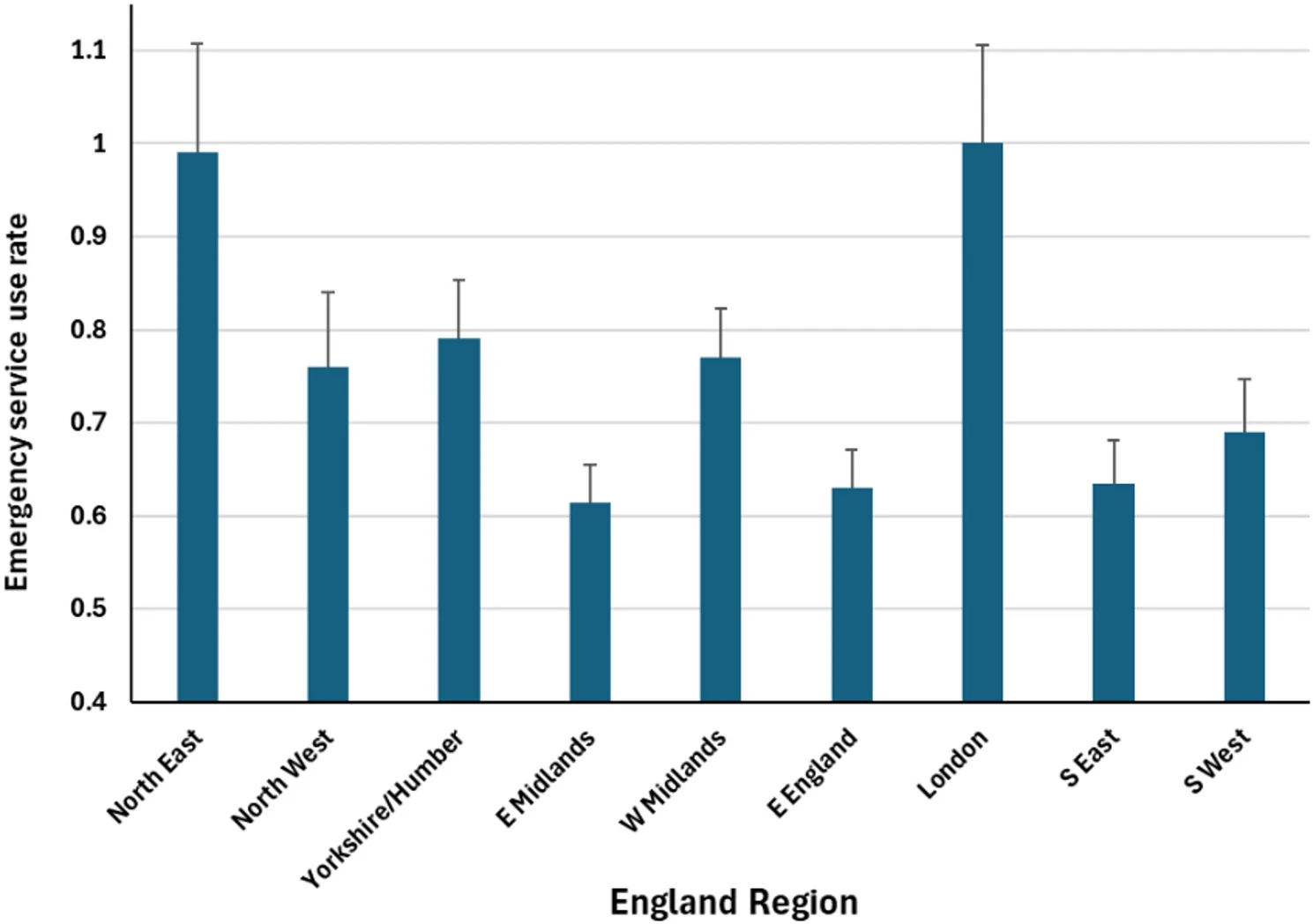
Regional differences of the emergency service use rate in children and young people with intellectual developmental disabilities (with 95% confidence interval error bars)

Overall, statistically significant UK regional differences were observed in the participant families use of emergency services (*F* = 2.418, *η*^2^= 0.024, *p* < 0.014, see Fig. 4). Children residing in London and the North East of England had significantly higher emergency use compared to the other regions, independent of individual-level factors. However, there were no significant differences between regions after Benjamini-Hochberg corrections (all *p*-values > 0.053).

#### 5. Genetic influence

There were no differences in the attendance rates between children with either genetic variant type [SNV: 0.76 visits per annum (95% CI = 0.75–0.78), CNV: 0.73 visits per annum (95% CI = 0.68–0.78), *t* = 0.621, *d* = 0.036, *p* = 0.535]; nor between those had an inherited versus a *de novo* variant [*De novo*: 0.70 visits per annum (95% CI = 0.63–0.77); inherited: 0.76 visits per annum (95% CI = 0.67–0.85), *t*=-1.308, *d* = 0.117, *p* = 0.191].

#### 6. Socioeconomic and sex influence

There were no differences in the frequency of attendance at emergency services between the sexes [Male = 0.73 visits per annum (95% CI = 0.66–0.80); Female = 0.74 visits per annum (95% CI = 0.66–0.82); *t* = 0.104, *d* = 0.007, *p* = 0.917]; or ethnicities (*F* = 4.041, η^2^ = 0.01, *p* = 0.40). There was also no relationship of between emergency service attendance and socioeconomic status as measured by IMD deciles (*F* = 1.002, η^2^ = 0.005, *p* = 0.406).

#### 7. Summary

Linear regression models show that age, state of general health, multimorbidity and region of residence were explanatory factors on children’s emergency service utilisation rate (see Model 11 and 12, other models in the Additional File 1: Table S4). Younger children had significantly higher rates of emergency utilisation [B(SE)=-0.02 (0.004), *p* < 0.001]. For every one-unit increase in age, the emergency use rate decreased by 0.02 visits/annum. Participants with a “very Bad” general health (by caregiver’s estimation) had higher rates of emergency visit than those with “Very Good” health [B(SE) = 0.072 (0.01), *p* < 0.001]. Children with multimorbidity had significantly higher emergency use rates [B(SE) = 0.052 (0.09), *p* < 0.001]. Those living in London had significantly higher rates of attendance than those living in East Midlands [B(SE)=-0.089 (0.041), *p* < 0.05] and East England B(SE)=-0.073 (0.035), *p* = 0.036]. Interestingly, there was a relationship with prosocial behaviour, so that for every one-unit increase in the prosocial score (measured by the SDQ), emergency service usage slightly decreased [B(SE)=-0.007 (0.003), *p* = 0.032].

## Discussion

### Overview

We investigated factors influencing emergency healthcare use among children and young people with intellectual developmental disabilities of genetic aetiology. Our participants had a wide range of congenital, neurodevelopmental, mental and physical health conditions. To our knowledge, this is the first study to consider A&E attendance in a nationally recruited cohort of children with IDD of genetic aetiology. We linked NHS records to primary carer-provided information on a range of possible explanatory factors. Participants used emergency services more than equivalently aged children from the general population. We found risk factors included younger age, impairment of adaptive functioning, more behavioural difficulties, less prosocial behaviour, poorer general health, and multisystem medical morbidity. In contrast, sex, ethnicity, socioeconomic deprivation (IMD), variant type, and inheritance status were not independently associated with utilisation in adjusted analyses. Regional variation was evident, with children residing in London and the North East experiencing significantly higher emergency use rates, a pattern that mirrors the regional trends observed in the general population.

### Age and developmental vulnerability

Younger age was associated with higher emergency use rates (see Additional File 1: Table S1). Early childhood is characterised by higher infection burden (such as respiratory and gastrointestinal illnesses), evolving neurological manifestations (e.g., seizure onset), and diagnostic uncertainty, which may account for this increased frequency of emergency attendance [35]. In general, unplanned emergency department return visits are significantly higher among infants and toddlers, particularly those with respiratory diseases and infections [36]. The presence of complex disability, combined with challenges in articulating symptoms, often leads to increased protective, risk-averse caregiving, particularly among parents of younger children [37, 38]. It is possible that improvements in preventive and community-based services could provide preventative support to families with children with IDD, to ameliorate same aspects of their need to access emergency services.

### Disability-related clinical complexity and emergency utilisation

Our findings support existing evidence that the excess of health needs that characterise many children with IDD are driven primarily by issues of clinical complexity and multimorbidity. Children with seizures, respiratory, cardiac, gastrointestinal, musculoskeletal, and endocrine-related conditions had markedly higher emergency utilisation rates. This aligns with prior UK evidence demonstrating elevated rates of unplanned hospital admissions and preventable mortality among individuals with learning disabilities, particularly those with epilepsy and multiple comorbid conditions [7, 10]. Importantly, emergency healthcare use in this population must be interpreted within the context of the natural history of these conditions. Seizures and chest problems, the top two clinical presentations within our cohort, frequently involve highly unpredictable, rapid escalations that necessitate immediate, acute emergency care as a matter of clinical safety. In cases of refractory epilepsy or severe lower respiratory tract infections, an emergency department visit is often an entirely appropriate and unpreventable consequence of underlying physiological frailty, rather than a failure of community care. Nevertheless, beyond these inherent clinical crises, emergency healthcare use in this population may also reflect both genuine acute medical need and potentially avoidable crises arising from fragmented care coordination. Children with complex, multisystem involvement often require highly coordinated multidisciplinary management. Gaps in continuity of care, limited specialist access, and insufficient anticipatory care planning may contribute to additional crisis-driven service use.

### Adaptive functioning and behavioural factors

Lower General Adaptive Composite (GAC) scores were independently associated with increased emergency utilisation. Adaptive functioning reflects daily living skills, communication, and social competence. Children with lower adaptive capacity may have reduced ability to communicate symptoms, engage in self-care, or tolerate illness-related stress, increasing caregiver reliance on emergency services.

Both externalising behaviour problems and poorer prosocial behaviour were associated with more frequent emergency service use in this cohort. This parallels the observation that hyperactivity and conduct problems are associated with increased emergency department utilisation in the general population [39–42], often through injury pathways [39, 40, 42]. Our surprising finding that prosocial behaviours protect against emergency clinic usage could reflect differences in social engagement, regulatory capacity, and behavioural stability. Poorer prosocial skills may increase caregiver stress and reduce coping capacity during illness episodes, potentially lowering thresholds for seeking emergency care. Previous research has shown that behavioural difficulties are associated with increased healthcare contact among children with neurodevelopmental conditions [24, 43], although few studies have specifically linked prosocial functioning to emergency utilisation.

These findings highlight the importance of integrating behavioural and adaptive assessments into risk stratification models for healthcare planning in children with IDD. Many children with complex needs do not receive well-coordinated, proactive care, and instead rely on reactive, crisis-driven services such as emergency departments [44–47].

### Regional variation and area-level factors

Significant regional differences were observed, with families living in London and the North East showing higher emergency utilisation rates. Those regional variations may reflect differences in health service configuration, primary care access, and population health profiles [18]. A large birth cohort study of emergency department use in England previously reported that emergency service attendance rates were highest in the North East (916 per 1,000 child-years) and London (876 per 1,000 child-years), compared with other regions in the general populations [29]. Our cohort’s results closely mirror the general population trends, highlighting how localised socio-demographic and structural pressures influence paediatric healthcare utilisation. The North East’s high emergency utilisation is strongly driven by area-level socioeconomic deprivation and high rates of childhood morbidity, while London’s spike reflects an overall demographic profile combined with a highly dense, ethnically diverse population and general practitioners access barriers, redirecting families toward emergency departments [15, 48, 49]. Our findings validate that emergency use is heavily shaped by these macro-level national healthcare dynamics rather than specific cohort anomalies.

Interestingly, our measures of socioeconomic status, the IMD decile, was not independently associated with emergency utilisation in adjusted models. This observation implies that, within a clinically complex IDD cohort, disability-related health needs overshadow area-level socioeconomic effects. Alternatively, regional service configuration and healthcare access factors may operate independently of individual deprivation status.

These findings underscore the need to examine structural and health system factors, including community paediatrics provision, epilepsy specialist access, and integrated care pathways, when addressing emergency healthcare disparities among children with complex disabilities of genetic aetiology.

### Genetic factors and healthcare utilisation

Variant type (SNV vs. CNV) and inheritance status were not significantly associated with emergency use rate. While genetic architecture contributes to phenotypic heterogeneity, healthcare utilisation in this cohort appears to be more strongly influenced by functional impairment and medical comorbidity than by variant classification or whether a parent is a carrier of the genetic anomaly. This finding suggests that service planning should prioritise specific factors related to the clinical profile and this vulnerable population require early support and monitoring [38].

### Implications for health equity and service design

This study highlights the central role of disability-related clinical complexity in shaping emergency healthcare demand. Strategies to reduce emergency utilisation among children with IDD of genetic aetiology should prioritise:

1. Proactive management of seizures and multisystem conditions, such as developing interoperable digital infrastructure – Incorporating automatic electronic alerts within Emergency Care Data Sets (ECDS) can immediately flag high-frequency attenders or children with complex medical histories upon arrival, triggering rapid and senior clinical triage [47];
2. Standardised health check screening following genetic diagnosis [50];
3. Better training for medical professionals to strengthen community paediatric and multidisciplinary coordination in the care of children and young people with IDD of genetic origin;
4. Early anticipatory care planning, including digitally accessible emergency care plan to ensure the general practitioners, ambulance crews and emergency department staff share the NHS connected care records [47, 51];
5. Screening for mental health issues among children with IDD of genetic aetiology; our study found up to 50% had associated neurodevelopmental disorders such as autism and attention deficit hyperactivity disorder;
6. Better information provision and support for caregivers managing children with exceptionally low adaptive functioning to increase the awareness of possible difficulties to expect and responses when they emerge.

### Region-specific service planning to address structural disparities

From a disability health equity perspective, emergency utilisation should not be interpreted as inappropriate service use. We present evidence that it is a potential indicator of unmet need and tends to reflect insufficient preventive infrastructure. Improving continuity of care and accessible specialist services may reduce avoidable emergency presentations while improving health outcomes.

### Strengths and limitations

Strengths of this study of the acute service needs of children with IDD include the application of data linkage from NHS Health Episode Statistics to a population-based cohort that had well-characterised genetic conditions. Also, the IMAGINE-ID study gathered multidimensional factors including adaptive, behavioural characteristics, primary caregiver-reported health measures and multimorbidity. Accordingly, it was possible to conduct a comprehensive assessment of factors affecting emergency service use. The longitudinal character of our study enabled us to investigate changes in the use of emergency services by age, allowing us to make a comparison across developmental stages.

There are several limitations. Firstly, some potentially important factors, such as whether families had access to or were supported by specific community healthcare services, were not captured in the dataset and may have influenced the observed associations. Secondly, we relied on caregiver-reported medical history for some variables. We lacked information about specific reasons for attendances at emergency department and consequently, some might have been avoidable, others not. Thirdly, lifetime emergency visits divided by age assumes constant risk over time and may not capture temporal clustering of utilisation. We know for instance that visits were less frequent by the time of adolescence but had been more frequent in the preschool period. Fourthly, on the 800 children for whom HES data were available, there were missing data (only 56.4% had GAC scores; 58% parents completed the medical questionnaire).

NHS England HES data cannot provide information about emergency care outside of England (such as in Wales or Scotland); some families lived close to national borders. Furthermore, our data collection window overlaps with the COVID-19 pandemic (2020–2022), a period known to have suppressed paediatric emergency department utilization across England. On the other hand, we did obtain statistics relating to cumulative lifetime emergency utilization rates spanning up to 15.5 years per participant, so most of our longitudinal data captures pre-pandemic behaviour. Finally, we obtained information from families at various time points throughout the longitudinal research project (2015–2025). Recruitment took place during the first 5 years of the project rather than at a uniform baseline prior to the tracking of healthcare utilization. Because the NHS England emergency department dataset spans the participants’ full lifetime histories, it is not methodologically possible to establish strict temporal precedence between specific survey responses and individual emergency presentations. Consequently, our findings must be interpreted strictly as cross-sectional associations rather than predictive or causal timelines. We cannot rule out the possibility of reverse causality or bidirectional effects; for example, a high cumulative lifetime frequency of acute emergency crises may itself contribute to poorer subsequent neurobehavioral functioning and heightened carer burden. Future longitudinal studies with tightly scheduled synchronized intervals of survey administration and healthcare data extraction are required to untangle these temporal dynamics.

## Conclusions

Among children with genetically confirmed intellectual developmental disabilities in England, emergency healthcare utilisation is primarily driven by younger age, reduced adaptive functioning, higher level of behavioural difficulties, lower prosocial behaviour, poorer general health, and multisystem co-morbidity. Socioeconomic deprivation and genetic factors were not independently associated with emergency use, whereas regional disparities were notable and reflected general population trends. Our findings emphasise that health inequalities among children with intellectual developmental disabilities of genetic aetiology are strongly linked to clinical complexity and system-level factors. Targeted, integrated, and regionally responsive healthcare strategies are required to reduce emergency service reliance and promote equitable health outcomes for children with IDD.

## Supplementary information

Below is the link to the electronic supplementary material.

Additional File 1

## Data Availability

The Hospital Episode Statistics data from NHS England that support the findings in this paper are not available for data sharing according to their guidelines. The IMAGINE-ID research data from this study are available from the authors upon application (see details of the application of data access in https://imagine-id.org/research-data-access/; contact person: Irene Lee, irene.lee@ucl.ac.uk).

## Abbreviations

ABAS-3: Adaptive Behaviour Assessment System 3
A&E: Accident & Emergency
CI: Confidence Interval
CNV: Copy Number Variant
CYP: Children and Young People
ECDS: Emergency Care Data Set
GAC: General Adaptive Composite
HES: Hospital Episode Statistics
IDD: Intellectual Developmental Disability
IMAGINE-ID: Intellectual Disability and Mental Health: Assessing the Genomic Impact on Neurodevelopment
IMD: Index of Multiple Deprivation
NHS: National Health Service
ONS: The Office for National Statistics
SD: Standard Deviation
SDQ: Strengths and Difficulties Questionnaire
SNV: Single Nucleotide Variant
UK: United Kingdom
χ2: Chi-Square
