# Supplementary material for: Factors associated with the use of UK emergency services in children and young people with intellectual developmental disabilities
Source: BMC Med. 2026 Oct 28;24:595. doi: 10.1186/s12916-026-05277-x (PMC13718101; doi:10.1186/s12916-026-05277-x)
Supplement: Supplementary file 1 [file 12916_2026_5277_MOESM1_ESM.docx]

**Additional Information**

**Factors associated with the use of UK emergency services in children and young people with intellectual developmental disabilities**

Irene O Lee^1,^*****, Jeanne Wolstencroft^1,2^, Harriet Housby^3^, Marianne B M van den Bree^4,5^, Samuel J R A Chawner^4^, Jeremy Hall^4,5^, Michael J Owen^4,5^, IMAGINE ID Consortium^6,^****** and David H Skuse^1^

^1^ Paediatric Mental Health Sciences Centre, Great Ormond Street Institute of Child Health, University College London, London, UK.

^2^ Department of Psychology, College of Health Medicine and Life Sciences, Brunel University of London, London, UK

^3^ The Division of Psychology and Language Sciences, Faculty of Brain Sciences, University College London, London, UK.

^4^ Centre for Neuropsychiatric Genetics and Genomics, Division of Psychological Medicine and Clinical Neurosciences, Cardiff University, Cardiff, UK.

^5^ Neuroscience and Mental Health Innovation Institute, Division of Psychological Medicine and Clinical Neurosciences, Cardiff University, Cardiff, UK.

^6^ IMAGINE ID Consortium, London, UK.

**IMAGINE ID Consortium, UK**

David H Skuse^1,^******

Jeanne Wolstencroft^1,2^

Irene O Lee^1^

Samuel Chawner^4^

Jeremy Hall^4,5^

Josh Hope-Bell^4^

Michael J Owen^4,5^

Marianne van den Bree^4,5^

F Lucy Raymond^6^

Ramya Srinivasan^7^

**** Consortium representative:** David H Skuse, Paediatric Mental Health Sciences Centre, Great Ormond Street Institute of Child Health, University College London, London, UK.

^1^ Paediatric Mental Health Sciences Centre, Great Ormond Street Institute of Child Health, University College London, London, UK.

^2^ Department of Psychology, College of Health Medicine and Life Sciences, Brunel University of London, London, UK

^4^ Centre for Neuropsychiatric Genetics and Genomics, Division of Psychological Medicine and Clinical Neurosciences, Cardiff University, Cardiff, UK.

^5^ Neuroscience and Mental Health Innovation Institute, Division of Psychological Medicine and Clinical Neurosciences, Cardiff University, Cardiff, UK.

^6^ Department of Medical Genetics, University of Cambridge, Cambridge, UK.

^7^ Division of Psychiatry, University College London, London, UK.

Josh Hope-Bell, F Lucy Raymond and Ramya Srinivasan did not directly contribute to the manuscript but were involved in other areas of the project.

**Table of Contents**

[**Note S1**. More information about the measures of the Strength and Difficulties Questionnaire (SDQ).	5](#_Toc241508272)

[**Note S2.** Medical history questions for the system-specific problems.	7](#_Toc241508273)

[**Table S1.** National Annual Emergency Department Attendance Rates in England.	8](#_Toc241508274)

[**Table S2.** Strength and Difficulties Questionnaire (SDQ) score and classification band of the cohort participants with intellectual developmental disabilities.	9](#_Toc241508275)

[**Table S3.** The number of cases and emergency visit rate of different combinations of system morbidity types in children and young people with intellectual developmental disabilities.	10](#_Toc241508276)

[**Table S4.** Linear regressions models of predictors for the emergency service utilisation rate in the cohort participants.	12](#_Toc241508277)

[**References**	14](#_Toc241508278)

# **Note S1**. More information about the measures of the Strength and Difficulties Questionnaire (SDQ).

The Strengths and Difficulties Questionnaire (SDQ) (Goodman, 1997) is a brief behavioural screening questionnaire about 2–17 year olds, and is a globally recognised instrument for assessing the mental health status for children and young people (Youth in Mind, 2016). SDQ scoring provides an overview to evaluate children and young people’s emotional and behavioural adjustment in dimensional terms (Goodman & Goodman, 2009; Murray et al., 2021).

There are 25 items in the SDQ which comprises of 5 scales in 5 items each that measure: emotional symptoms; conduct problems; hyperactivity, impulsivity, and inattention difficulties; peer relationship problems; and prosocial behaviour. The first four of these scales are combined to make a total difficulties score. Higher scores are indicative of a greater mental health difficulty and scores above 90th percentile indicate a high probability of a diagnosable psychiatric disorder; whereas a lower score on the prosocial behaviour implies greater difficulties (Goodman & Goodman, 2009; Goodman, 2001). The SDQ has been used as a standardised assessment to evaluate emotional and behavioural difficulties of children and adolescents with intellectual and developmental disabilities (Murray et al., 2021).

The SDQ scores were categorised into four bands based on a general population UK survey: 80% of UK children score in a “Close to Average” range regarded as ‘normal’, 10% score “Slightly Raised” range as ‘borderline’, and 10% score “High” or “Very High” range regarded as ‘abnormal’ (Youth in Mind, 2016).

Categorisation bands for SDQ scores are listed in the following table to identify the correct classification for each score.

For Parent/Carer completed SDQ:

| **Classification for each SDQ subscale score** | **Close to**  **Average**  (80% pop) | **Slightly raised**  **(/lowered)**  (10% pop) | **High**  **(/Low)**  (5% pop) | **Very high**  **(/very low)**  (5% pop) |
| --- | --- | --- | --- | --- |
| Emotional problems score | 0-3 | 4 | 5-6 | 7-10 |
| Conduct problems score | 0-2 | 3 | 4-5 | 6-10 |
| Hyperactivity score | 0-5 | 6-7 | 8 | 9-10 |
| Peer problems score | 0-2 | 3 | 4 | 5-10 |
| Prosocial score | 8-10 | 7 | 6 | 0-5 |
| **Total difficulties score** | 0-13 | 14-16 | 17-19 | 20-40 |

Note: pop=population.

For further information regarding the scoring process, please visit: <https://sdqscore.org/>.

**Note S2.** Medical history questions for the system-specific problems.

The primary caregivers had given choices of “yes” or “no” for each question if the children and young people of their care had the system-specific problems.

1. **Auditory:** Has your child ever had any hearing problems?
2. **Vision:** Has your child ever had any eye problems?
3. **Clef palate:** Has your child ever had a cleft lip or palate?
4. **Muscle or movement**: Has your child ever had any muscle or movement problems?
5. **Cranial:** Has your child ever had any brain damage caused by an infection or trauma?
6. **Seizures:** Has your child ever had any seizures?
7. **Gastrointestinal:** Has your child ever had any stomach and/or gut problems?
8. **Respiratory:** Has your child ever had any breathing or chest problems?
9. **Cardiac:** Has your child ever had any heart problems?
10. **Renal:** Has your child ever had any kidney problems?
11. **Genital:** Has your child ever had any genital problems?
12. **Bone/Skeletal:** Has your child ever had any skeletal or bone problems?
13. **Growth/Hormone**: Has your child ever had any growth or hormone problems?
14. **Sleep:** Has your child ever had any sleep problems?

**Table S1.** National Annual Emergency Department Attendance Rates in England.

| **Age group (years)** | **Annua Attendance Rate (per 1000 people)** | **Average Visits per Person (per annum)** | **Primary Clinical Drivers** |
| --- | --- | --- | --- |
| 0-4 | ~600-795 | 0.60-0.80 | Acute infections, high parental anxiety, sudden fevers |
| 5-14 | ~184 to 250 | 0.18-0.25 | Sports injuries, accidental trauma, fractures |
| 15-34 | ~345 to 425 | 0.35-0.42 | Acute injuries, alcohol/ substance-related incidents, mental health crisis |
| 65-79 | ~334 to 391 | 0.33-0.39 | Exacerbation of chronic conditions, falls, cardio-respiratory events |
| 80+ | ~692 to 842 | 0.69-0.84 | Severe frailty, multi-morbidity, acute infections, system failures |

Data source from: 1. Annual Report Series, datasets from Hospital Accident & Emergency Activity, 2022–23 through to more recent annual updates) (NHS-England, 2023). 2) Database of Hospital Episode Statistics (HES): Accident and Emergency (NHS-England, 2026).

**Table S2.** Strength and Difficulties Questionnaire (SDQ) score and classification band of the cohort participants with intellectual developmental disabilities.

| **SDQ score** | **Mean ± SD (95% CI)** | **Band** |
| --- | --- | --- |
| **Total Difficulties** | 20.3 ± 6.8 (19.8-20.8) | Very High |
| **Emotional Problems** | 4.5 ± 2.9 (4.3-4.7) | Slightly Raised |
| **Conduct Problems** | 3.5 ± 2.4 (3.3-3.7) | Slightly Raised |
| **Hyperactivity** | 7.9 ± 2.1 (7.7-8.1) | Slightly Raised |
| **Peer Problems** | 4.4 ± 2.3 (4.2-4.6) | High |

SD=Standard deviation; CI=Confidence interval.

**Table S3.** The number of cases and emergency visit rate of different combinations of system morbidity types in children and young people with intellectual developmental disabilities.

| **Combination of system morbidity type** | **Count of cases** | **Average emergency visits per annum** | **SD** |
| --- | --- | --- | --- |
| Seizure+Chest+Heart+Growth | 1 | 2.74 | NA |
| Seizure+Chest+Bone | 3 | 2.17 | 1.88 |
| Seizure+Chest+Heart+Bone+Growth | 2 | 1.62 | 0.95 |
| Seizure+Gut+Heart | 2 | 1.62 | 0.21 |
| Seizure+Gut+Bone | 5 | 1.61 | 0.75 |
| Seizure+Growth | 3 | 1.59 | 1.53 |
| Seizure+Gut+Chest+Heart+Bone | 2 | 1.42 | 1.51 |
| Gut+Chest+Growth | 8 | 1.38 | 1.03 |
| Seizure+Gut+Chest+Growth | 5 | 1.37 | 0.66 |
| Seizure+Gut+Chest | 8 | 1.34 | 1.35 |
| Chest+Heart+Bone | 5 | 1.29 | 1.07 |
| Gut+Chest+Heart+Bone+Growth | 4 | 1.28 | 0.88 |
| Gut+Chest+Heart | 15 | 1.27 | 0.79 |
| Seizure+Gut+Chest+Heart+Bone+Growth | 3 | 1.26 | 0.56 |
| Gut+Heart | 1 | 1.24 | NA |
| Gut+Chest+Heart+Growth | 4 | 1.20 | 0.54 |
| Seizure+Gut+Heart+Bone | 3 | 1.18 | 0.34 |
| Seizure+Heart | 7 | 1.16 | 1.17 |
| Chest+Heart+Growth | 5 | 1.02 | 0.68 |
| Seizure+Chest+Growth | 1 | 1.02 | NA |
| Seizure+Chest+Heart+Bone | 1 | 1.01 | NA |
| Seizure+Chest+Heart | 6 | 0.93 | 0.63 |
| Seizure+Gut+Chest+Bone | 3 | 0.92 | 0.92 |
| Chest+Bone+Growth | 1 | 0.88 | NA |
| Seizure+Gut+Heart+Growth | 1 | 0.84 | NA |
| Seizure+Gut+Chest+Heart | 3 | 0.80 | 0.04 |
| Chest+Heart | 13 | 0.80 | 0.76 |
| Chest+Bone | 4 | 0.79 | 0.76 |
| Seizure+Gut+Bone+Growth | 1 | 0.78 | NA |
| Seizure+Gut | 10 | 0.74 | 0.58 |
| Seizure+Chest | 13 | 0.70 | 0.39 |
| Gut+Heart+Bone | 3 | 0.68 | 0.59 |
| 0 | 432 | 0.68 | 0.79 |
| Chest | 41 | 0.68 | 0.63 |
| Seizure | 32 | 0.68 | 0.54 |
| Gut+Bone+Growth | 5 | 0.67 | 0.63 |
| Gut+Bone | 3 | 0.66 | 0.59 |
| Seizure+Bone+Growth | 2 | 0.66 | 0.66 |
| Chest+Heart+Bone+Growth | 3 | 0.65 | 0.58 |
| Heart+Growth | 5 | 0.64 | 0.39 |
| Seizure+Gut+Chest+Heart+Growth | 1 | 0.62 | NA |
| Gut+Chest | 22 | 0.58 | 0.39 |
| Gut+Chest+Bone | 5 | 0.56 | 0.26 |
| Growth | 8 | 0.55 | 0.76 |
| Gut | 44 | 0.55 | 0.45 |
| Gut+Growth | 14 | 0.53 | 0.34 |
| Heart+Bone | 1 | 0.49 | NA |
| Seizure+Gut+Chest+Bone+Growth | 1 | 0.48 | NA |
| Seizure+Gut+Growth | 2 | 0.48 | 0.26 |
| Bone | 7 | 0.45 | 0.28 |
| Gut+Chest+Heart+Bone | 1 | 0.44 | NA |
| Heart | 14 | 0.42 | 0.37 |
| Chest+Growth | 4 | 0.42 | 0.08 |
| Seizure+Bone | 2 | 0.34 | 0.27 |
| Gut+Chest+Bone+Growth | 1 | 0.32 | NA |
| Seizure+Gut+Heart+Bone+Growth | 1 | 0.25 | NA |
| Bone+Growth | 1 | 0.20 | NA |
| Gut+Heart+Growth | 1 | 0.17 | NA |
| Heart+Bone+Growth | 1 | 0.08 | NA |

SD = standard deviation; NA=not applicable.

**Table S4.** Linear regressions models of predictors for the emergency service utilisation rate in the cohort participants.

| **Regression Model** | **B (SE)** | ***Beta*** | ***p*-value** |
| --- | --- | --- | --- |
| **Model 1 (N=800)**  Age | R^2^=5.5%  -0.024 (0.003) | -0.237 | *<0.001* |
| **Model 2 (N=800)**  Sex  Female *(Male as reference)* | R^2^=0.1%  -0.002 (0.02) | -0.004 | 0.917 |
| **Model 3 (N=800)**  Variant type  CNV *(SNV as reference)* | R^2^=0.1%  -0.013 (0.02) | -0.022 | 0.535 |
| **Model 4 (N=530)**  Genetic Inheritance  Familial (*De novo* as reference) | R^2^=0.1%  0.001 (0.002) | 0.013 | 0.722 |
| **Model 5 (N=717)** | R^2^=8.4% |  |  |
| General health  *(Very Good health as reference)* | 0.079 (0.01) | 0.291 | *<0.001* |
| **Model 6 (N=475)**  Number of multiple morbidities | R^2^=12.4%  0.064 (0.01) | 0.355 | *<0.001* |
| **Model 7 (N=451)**  GAC standard score | R^2^=1.2%  -0.002 (0.001) | -0.121 | *0.010* |
| **Model 8 (N=738)**  Total Difficulties score  Hyperactivity score  Prosocial score | R^2^=1.8%  0.002 (0.002)  0.002 (0.006)  -0.010 (0.003) | 0.053  0.014  -0.118 | 0.276  0.770  *0.002* |
| **Model 9 (N=796)**  Index of Multiple Deprivation | R^2^=0.1%  -0.005 (0.003) | -0.061 | 0.084 |
| **Model 10 (N=800)** | R^2^=7.0% |  |  |
| *(London as reference)* |  |  |  |
| North East | 0.042 (0.053) | 0.032 | 0.427 |
| North West | -0.038 (0.04) | -0.043 | 0.344 |
| Yorkshire and Humber | -0.029 (0.035) | -0.040 | 0.420 |
| East Midlands | -0.075 (0.041) | -0.079 | 0.073 |
| West Midlands | -0.033 (0.035) | -0.046 | 0.355 |
| East England | -0.088 (0.035) | -0.124 | *0.013* |
| South East | -0.086 (0.033) | -0.134 | *0.010* |
| South West | -0.067 (0.04) | -0.076 | 0.094 |
| **Model 11 (N=427)** | R^2^=19.1% |  |  |
| Age | -0.021 (0.004) | -0.207 | *<0.001* |
| General health | 0.041 (0.013) | 0.153 | *0.002* |
| Number of multiple morbidities | 0.052 (0.009) | 0.290 | *<0.001* |
| GAC Standard score | -0.001 (0.001) | -0.042 | 0.348 |
|  |  |  |  |
| **Model 12 (N=715)** | R^2^=13.1% |  |  |
| Age | -0.019 (0.004) | -0.187 | *<0.001* |
| General health | 0.072 (0.01) | 0.267 | *<0.001* |
| Prosocial score | -0.007 (0.003) | -0.078 | *0.032* |
| (London as reference) |  |  |  |
| North East | 0.054 (0.052) | 0.042 | 0.298 |
| North West | -0.023 (0.039) | -0.027 | 0.556 |
| Yorkshire and Humber | -0.034 (0.035) | -0.049 | 0.330 |
| East Midlands | -0.080 (0.041) | -0.086 | *0.049* |
| West Midlands | -0.035 (0.035) | -0.05 | 0.318 |
| East England | -0.073 (0.035) | -0.106 | *0.036* |
| South East | -0.064 (0.033) | -0.101 | 0.054 |
| South West | -0.050 (0.039) | -0.057 | 0.207 |
|  |  |  |  |

N=Number of cases; R^2^=Adjusted R^2^; B=Unstandardized coefficient; SE=Standard error; *Beta*=Standardized coefficients.

**References**

Goodman, A., & Goodman, R. (2009). Strengths and Difficulties Questionnaire as a Dimensional Measure of Child Mental Health. *Journal of the American Academy of Child and Adolescent Psychiatry*, *48*(4), 400-403. <https://doi.org/10.1097/CHI.0b013e3181985068>

Goodman, R. (1997). The Strengths and Difficulties Questionnaire: a research note. *J Child Psychol Psychiatry*, *38*, 581–586. <https://doi.org/https://acamh.onlinelibrary.wiley.com/doi/epdf/10.1111/j.1469-7610.1997.tb01545.x>

Goodman, R. (2001). Psychometric properties of the strengths and difficulties questionnaire. *J Am Acad Child Adolesc Psychiatry*, *40*, 1337-1345. <https://doi.org/10.1097/00004583-200111000-00015>

Murray, C. A., Hastings, R. P., & Totsika, V. (2021). Clinical utility of the parent-reported Strengths and Difficulties Questionnaire as a screen for emotional and behavioural difficulties in children and adolescents with intellectual disability. *The British Journal of Psychiatry*, *218*, 323–325. <https://doi.org/10.1192/bjp.2020.224>

NHS-England. (2023). *Hospital Accident & Emergency Activity, 2022-23. NHS Digital, 2023.* <https://digital.nhs.uk/data-and-information/publications/statistical/hospital-accident--emergency-activity/2022-23>

NHS-England. (2026). *A&E Attendances and Emergency Admissions*. <https://www.england.nhs.uk/statistics/statistical-work-areas/ae-waiting-times-and-activity/>

Youth in Mind. (2016). *Scoring the Strengths and Difficulties Questionnaire for ages 4–17. In Scoring the SDQ*. Youth in Mind. <https://www.sdqinfo.org/py/sdqinfo/c0.py>
